# A rapid and efficient way to manage hyponatremia in patients with SIADH and small cell lung cancer: treatment with tolvaptan

**DOI:** 10.1186/1471-2466-13-55

**Published:** 2013-08-29

**Authors:** Claudia Petereit, Okan Zaba, Ishak Teber, Heike Lüders, Christian Grohé

**Affiliations:** 1 ELK Department of Respiratory Diseases, Lindenberger Weg 27, 13125, Berlin, Germany

**Keywords:** Small cell lung cancer (SCLC), Hyponatremia, Syndrome of inappropriate antidiuretic hormone hypersecretion (SIADH)

## Abstract

**Background:**

Hyponatremia based on syndrome of inappropriate antidiuretic hormone hypersecretion (SIADH) is observed in up to 15% of patients with small cell lung cancer (SCLC). The electrolyte imbalance is associated with a high morbidity and mortality and often delays appropriate treatment. Management of hyponatremia proved to be challenging until new vasopressin-2 receptor antagonists such as tolvaptan became available. This is the first report which presents a prospective case series with an efficient management of hyponatremia including tolvaptan in ten patients with SCLC and severe SIADH (plasma sodium < 125 mmol/l).

**Methods:**

Ten patients with SCLC and severe SIADH were followed after the onset of clinical symptoms of SIADH. Patients were chosen on the basis of histological proven diagnosis of SCLC and the clinical picture of a neurocognitive deficit caused by SIADH-related hyponatremia. All patient data were monitored for clinical improvement based on ECOG status, commencement of chemotherapy and correction of sodium levels.

**Results:**

The treatment followed a diagnostic and treatment algorithm and lead to a rapid and efficient correction of both clinical symptoms and plasma sodium level.

**Conclusions:**

Based on this algorithm all patients started chemotherapy in time. Subsequently, the treatment with tolvaptan lead to an improvement of the ECOG-performance status. In addition, all patients benefit from the effective management of SIADH which omitted prolonged hospital stays and non-elective hospitalizations due to an unstable clinical condition due to severe hyponatremia. These observations add new insight to management of SIADH in thoracic oncology and are of interest for specialists in oncology, endocrinology and pulmonary medicine.

## Background

Lung cancer is one of the leading causes of death for cancer-related mortality worldwide. In 2007 1.3 million people were diagnosed with lung cancer. Small cell lung cancer (SCLC) is found in up to 15–20% of all newly diagnosed lung cancer cases [1,2]. The highly malignant SCLC tumor consists of primitive cells derived from the neuroendocrine lung and is associated with paracrine and paraneoplastic syndromes. Smoking, sex, initial tumor burden and LDH levels have been found to play a role in the outcome and prognosis of the patients. As recently demonstrated, persistent hyponatremia also appears to be a negative prognostic marker for overall survival in SCLC patients [3-5].

Hyponatremia is common in patients with lung cancer [6-8]. Clinical symptoms such as dizziness, tremor, agitation and other neuropsychiatric symptoms have to be taken into consideration for the diagnosis of clinically relevant hyponatremia. Mild symptoms such as fatigue occur in mild (plasma sodium = 130-134 mmol/l) and in moderate hyponatremia (plasma sodium =125-129 mmol/l) [9]. Clinical symptoms can aggravate in hyponatremia (plasma sodium <125 mmol/l). The SIADH is a common finding in SCLC patients with euvolemic hyponatremia [10].

SIADH as a hallmark of SCLC can be found in 10-15% of all SCLC patients, but only in 2-4% of all non-small cell lung (NSLC) cancer patients, as recent retrospective series demonstrated [3,4,11]. In SCLC patients SIADH is diagnosed predominantly in advanced stages and may induce a significant reduction in plasma sodium levels. Retrospective data point out that hyponatremia and the lack of correction of sodium can lead to a poor outcome in SCLC patients. Furthermore, these data suggest that the correction of hyponatremia may affect the prognosis of the patients [3,4]. Correction and stabilisation of the sodium levels are required to initiate chemotherapy treatment and finally for the successful treatment of the underlying disease.

At the time of diagnosis SCLC patients in advanced stages of disease are often in a very limited performance status. In addition, comorbidities such as hyponatraemia or cardiovascular disease may delay the initiation of systemic chemotherapy and increase early mortality. Historical cohorts have shown that up to 40% of patients will not receive an effective primary chemotherapy due to a low Karnofsky-Index [2]. New, supportive therapeutic options such as the rapid stabilization of the sodium metabolism could be helpful in this context.

However, no prospective studies for the treatment of hyponatremia in SCLC patients have been reported on a larger scale, due to the lack of a standardized hyponatremia therapy. Recently, the vasopressin-2-receptor antagonist tolvaptan has been approved for the treatment of severe SIADH-associated hyponatremia [12]. An efficient correction of hyponatremia by tolvaptan has been found in a subgroup of SIADH patients out of mixed etiologies [12]. New data has shown that intervention with tolvaptan may be effective for correction and stabilisation of acute onset hyponatremia in SIADH patients [5,13].

Up to now, only few data exist for SCLC patients with SIADH and sodium levels below 125 mmol/l and no data showing a long term follow-up of treatment with tolvaptan. This analysis shows a prospective case series for hyponatremia management with tolvaptan in ten SCLC patients with severe SIADH.

## Methods

In ten patients with SCLC and severe SIADH (pNa < 125 mmol/l), diagnosed in the Evangelische Lungenklinik Berlin between June 2010 and April 2012, HN treatment including tolvaptan was monitored. An ethics committee approval was sought and granted (EV.-No.EA1-289-09). All patients provided consent for publication of individual clinical details in this manuscript.

SIADH was confirmed by the measurement of plasma and urine sodium and plasma and urine osmolality. Hypo- and hypervolemic hyponatremia were excluded as well as hyponatremia due to other endocrine causes such as adrenal insufficiency based on adrenal gland metastasis or hypothyroidism. Hyponatremia-inducing medication such as thiazides or carbamazepine were discontinued.

**Figure 1:**
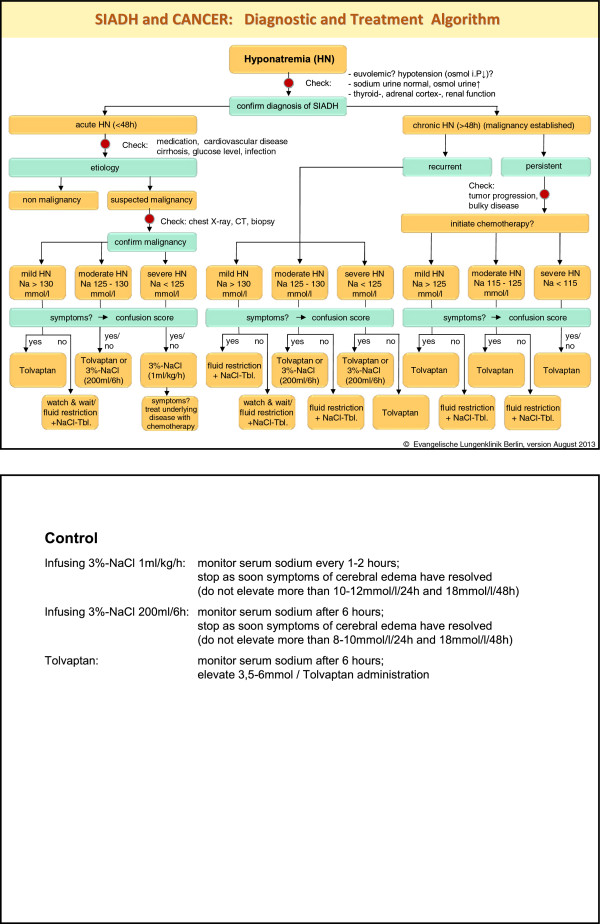
Diagnostic and treatment algorithm.

For management of hyponatremia, a diagnostic and treatment algorithm was designed (see Figure 1). All patients with hyponatremia were treated according to this algorithm.

When standard hyponatremia treatment such as fluid restriction or 3% hypertonic saline infusions failed to improve the clinical condition, patients received an initial dose of 15 mg/d tolvaptan. Alternative hyponatremia treatment options were stopped 12 hours beforehand. During tolvaptan treatment, no fluid restriction was enforced.

All patients with tolvaptan treatment were monitored as inpatients. Weight and the clinical status were assessed frequently and the clinical and laboratory data were registered and analyzed. The ECOG performance status was surveyed as a scale for quality of life. Follow-up data were collected for outcome analyses. Deadline for follow-up was December, 31 in 2012.

## Results

The patient characteristics in Table 1 show that most SCLC patients were in stage IV. Gender and age distributions were within standard deviations. In eight of ten patients, SIADH has been found in time of diagnosis (see Diagrams 1 to 8). Two of ten patients developed SIADH during the course of the disease (see Diagrams 9 and 10).

**Table 1:** Patients characteristics for ten monitored SCLC patients with confirmed SIADH

**Case**	**Gender**	**Age at diagnosis (years)**	**Sodium level at therapy start (mmol/l)**	**TNM**	**Overall survival (months)**
1	m	52	121	T4 N1 M1b (brain)	19
2	m	70	121	T4 N3 M1b (hepatic, osseous, pleura)	8
3	m	66	118	T4 N3 M1b (cervical, pulmonal, osseous)	8
4	f	62	115	T4 N2 M0	20, lives
5	f	62	118	T4 N3 M1b (hepatic)	11
6	m	66	120	T3 N2 M0	11
7	m	50	116	T2a N1 M0	2
8	f	63	117	T4 N3 M0	11, lives
9	m	71	122	T4 N3 M1a	5
10	m	65	117	T4 N3 M1b (hepatic, osseous)	10

(Localisation of the metastases indicated in parentheses).

In all patients with SCLC and SIADH management lead to a rapid clinical improvement. Platinum based chemotherapy could be administered in all patients.

**Figure 2:**
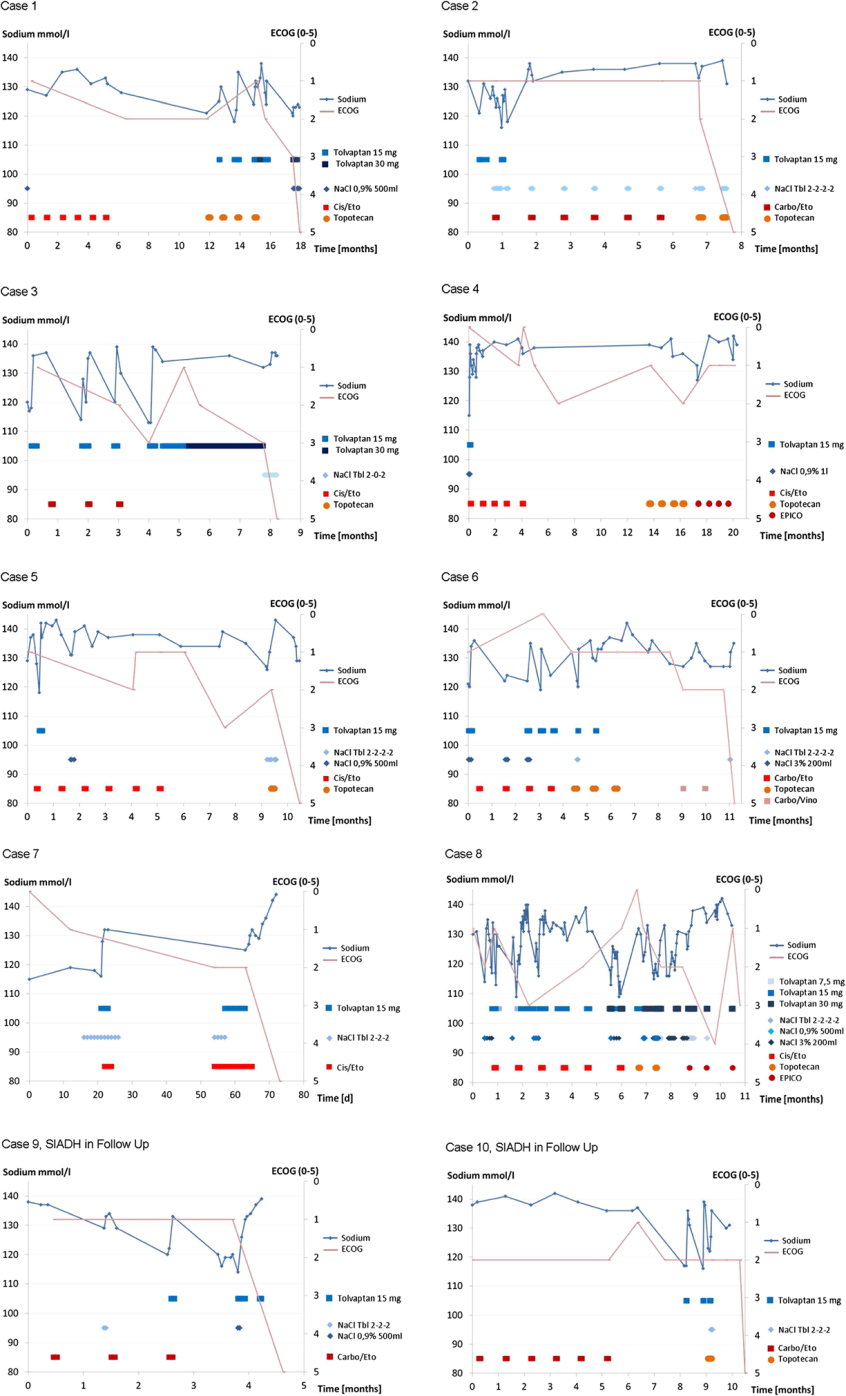
Diagrams for the cases one to ten with the clinical course (sodium levels, applied chemotherapy, ECOG performance status).

The dose of 15 mg tolvaptan per day was sufficient to raise the plasma sodium levels significantly. The median number of days of tolvaptan treatment per treatment episode was 4 days (an episode was defined as continuously given treatment up to an interruption >72 hours). Only two patients needed long-term tolvaptan treatments > 10 days to stabilize the plasma sodium levels > 125 mmol/l. The duration of plasma sodium maintenance >125 mmol/l was in median 17.5 days (range 2 to 614 days), as shown in Table 2 (Figure 2).

**Table 2:** Number of tolvaptan epissodes, days of tolvaptan treatment and duration of pNa maintenance for the ten monitored SCLC patients with confirmed SIADH

**Case**	**Number of tolvaptan episodes**	**Days of tolvaptan treatment per episode**			**Duration of pNa maintenance >125 mmol/l per episode [days]**		
		**Median**	**Min**	**Max**	**Median**	**Min**	**Max**
1	8	4	1	6	6	4	49
2	3	3	1	3	11	4	199
3	5	7	5	102	14	3	45
4	1	4	4	4	614	614	614
5	1	4	4	4	301	301	301
6	6	2	2	6	25	12	177
7	2	5	3	7	22	10	34
8	15	4	1	18	11	2	29
9	3	3	2	4	16	8	24
10	3	2	2	2	19	5	22
**Total**	**47**	**4**	**1**	**102**	**17,5**	**2**	**614**

No serious adverse events or neuropsychiatric deficits were observed during treatment with tolvaptan or in the post-interventional clinical course. In particular, in cases with rapid increase of sodium levels > 10 mmol/l in 24 hours, we found no neurological deficits. Treatment with tolvaptan was accompanied by the improvement of the ECOG performance status and ensured that 1st and 2nd line chemotherapy options were commenced in all patients.

In patients with tolvaptan treatment, no non-elective admissions due to clinical worsening or prolonged hospital stays were identified as the cause of persistent hyponatremia.

The follow-up time until death was 2–20 months (median 10, 5 months); two patients are alive. None of the patients died due to the hyponatremia intervention.

## Discussion

SCLC belongs to a class of the most malignant solid tumours. At the time of diagnosis most of the patients (90%) have to be classified as patients with limited, palliative treatment options. Due to the advanced age of this cohort (median 70 years) comorbidities such as cardiovascular diseases or respiratory insufficiency influence the therapeutic strategies. Paraneoplastic syndromes, hyponatremia in particular, is common in SCLC (up to 15% of all patients). In 1957, Schwartz and Bartter described this clinical phenomenon of inappropriate antidiuretic hormone (ADH) secretion in patients with lung cancer and introduced the term SIADH.

In general, SIADH can be diagnosed on the basis of plasma and urine sodium levels and osmolality. Treatment of SIADH should always be set in correlation with the clinical picture of hyponatremia before the treatment option is chosen. Chronic hyponatremia (plasma Na < 125 mmol/l) in lung cancer can often be oligosymptomatic. Acute loss of sodium in hyponatremia is, in contrast, associated with agitation, lack of concentration or compliance and requires urgent control of the symptoms. Therefore, before treatment options are chosen in individual cases, and especially in clinical presentations such as fatigue or wasting syndrome, the concerted action of specialists in the field such as endocrinologists and pulmonologists is sought.

As Hansen et al. [4] pointed out, hyponatremia in lung cancer patients is not only confined to the diagnosis of paraneoplastic SIADH. Other causes such as co-medications should be checked and considered to optimize the therapeutic goal. Hyponatremia treatment is based on clinical and biochemical parameters. The diagnostic and treatment algorithm given in Figure 1 proved to be helpful for a standardization of the hyponatremia management.

SIADH in SCLC is up to now a therapeutic challenge. Treatments with conventional therapeutic options such as fluid restriction or sodium chloride substitution, as well as with oral or intravenous medications, are often insufficient. Therapeutic oral drug preparations, such as demeclocycline, show only a slow and delayed increase in sodium levels in SIADH-associated hyponatremia. Fluid restriction or 3% hypertonic saline infusions is left as an option to reduce clinical symptoms which are required to start treatment. As most patients were in stage IV, an early commencement of chemotherapy was sought. A poor control of hyponatremia in this cohort may induce delays in the initiation of chemotherapy and consequently raise the disease-related morbidity and possibly mortality.

As illustrated in this analysis for SCLC patients with severe SIADH for the first time, hyponatremia management with tolvaptan lead to a rapid correction and stabilization of the plasma sodium level and can enable patients to receive chemotherapy. Subsequently, the treatment with tolvaptan supported the treatment of the underlying SCLC disease and lead to an amelioration of the ECOG-performance status. In all patients studied, no neurological deficits were observed. In particular, no clinical presentations of sodium overcorrection, as has been reported with hypertonic saline infusion, which may cause central pontine myelinolysis, were observed.

## Conclusion

Short treatment periods of tolvaptan (15 mg/d) were sufficient to stabilize sodium levels in the majority of the cases. Correction of sodium and the amelioration of the clinical symptoms may help to improve the prognosis in patients with extensive disease. In addition, hyponatremia management may shorten inpatient treatment periods and minimize emergency room calls. Furthermore, treatment with tolvaptan is a recommendable and safe treatment option for terminally ill patients who require emergency treatment in a palliative situation.

Further studies are necessary to analyze whether lower doses of tolvaptan (<15 mg/d) are sufficient for an effective treatment of severe SIADH in SCLC, as these patients responded very efficient to the dose of 15 mg/d. It is reasonable to imagine that tolvaptan may become a standard treatment option in primary or recurrent SCLC patients with SIADH, especially in patients with bulky disease and severe hyponatremia.

## Competing interests

No application for any patents relating to the content of the manuscript are declared.

OZ and CG have received reimbursements for case presentation from Otsuka, Pharmaceuticals, Inc.

The institution was supported by an unrestricted grant. No other financial competing interests are declared.

Non-financial competing interests: There are no any non-financial competing interests (political, personal, religious, ideological, academic, intellectual, commercial or any other) to declare in relation to this manuscript.

## Authors’ contribution

Conception and Design CG. Monitoring of Patients CP, IT, OZ, CG. Manuscript Writing CG, HL. Preparation of figures and tables CP, HL, CG. Final Approval of Manuscript CP, IT, OZ, HL, CG. All authors read and approved the final manuscript.

## Pre-publication history

The pre-publication history for this paper can be accessed here:

http://www.biomedcentral.com/1471-2466/13/55/prepub

## References

[B1] Van Meerbeeck JP Fennell DA De Ruysscher DK Small-cell lung cancer Lancet 2011 378 9804 1741 55 10.1016/S0140-6736(11)60165-7 21565397

[B2] Kalemkerian GP Akerley W Bogner P Borghaei H Chow L Downey RJ Gandhi L Ganti AK Govindan R Grecula JC Hayman J Heist RS Horn L Jahan TM Koczywas M Moran CA Niell HB O’Malley J Patel JD Ready N Rudin CM Williams CC Jr Small cell lung cancer National Comprehensive Cancer Network 2011 10 1086 113 10.6004/jnccn.2011.0092 21975911

[B3] Petereit C Zaba O Teber I Grohé C Is hyponatremia a prognostic marker of survival for lung cancer? Pneumologie 2011 65 9 565 71 21837588 10.1055/s-0030-1256668

[B4] Hansen O The occurrence of hyponatremia in SCLC and the influence on prognosis. A retrospective study of 453 patients treated in a single institution in a 10 year period Lung Cancer 2010 68 111 114 10.1016/j.lungcan.2009.05.015 19535164

[B5] Iyer AV Sodium wasting nephropathy caused by cisplatinum in a patient with small cell lung cancer Clin Lung Cancer 2003 5 187 189 10.3816/CLC.2003.n.033 14667276

[B6] Castillo JJ Vincent M Justice E Diagnosis and management of hyponatremia in cancer patients Oncologist 2012 17 756 65 10.1634/theoncologist.2011-0400 22618570 PMC3380874

[B7] Renneboog B Mild chronic hyponatremia is associated with falls, unsteadiness, and attention deficits Am J Med 2006 119 1 1 8 10.1016/j.amjmed.2005.06.050 16431193

[B8] Schwartz WB A syndrome of renal sodium loss and hyponatremia probably resulting from inappropriate secretion of antidiuretic hormone Am J Med 1957 23 529 542 10.1016/0002-9343(57)90224-3 13469824

[B9] Sorensen JB Syndrome of inappropriate secretion of anitdiuretic hormone (SIADH) in malignant disease J Inten Med 1995 238 97 110 10.1111/j.1365-2796.1995.tb00907.x 7629492

[B10] Verbalis JG Adler S Schrier RW Berl T Zhao Q Czerwiec FS Efficacy and safety of oral tolvaptan therapy in patients with the syndrome of inappropriate antidiuretic hormone secretion SALT Investigators. Eur J Endocrinol 2011 164 5 725 32 10.1530/EJE-10-1078 PMC3573862 21317283

[B11] Kenz S Haas CS Werth SC Bohnet S Brabant G High sensitivity to tovaptan in paraneoplastic syndrome of inappropriate ADH secretion (SIADH) Ann Oncol 2011 22 12 2696 22021821 10.1093/annonc/mdr431

[B12] Vanhees SL Paridaens R Vansteenkiste JF Syndrome of inappropriate antidiuretic hormone associated with chemotherapy-induced tumour lysis in small-cell lung cancer: case report and literature review Ann Oncol 2000 11 8 1061 5 10.1023/A:1008369932384 11038047

[B13] Onitilo AA Tumor related hyponatremia Clin Med Res 2007 5 4 228 237 10.3121/cmr.2007.762 18086907 PMC2275758

